# Social cognitive neuroscience in the digital age

**DOI:** 10.3389/fnhum.2023.1168788

**Published:** 2023-05-30

**Authors:** Margaret M. Doheny, Nichole R. Lighthall

**Affiliations:** Department of Psychology, University of Central Florida, Orlando, FL, United States

**Keywords:** social cognition, cognitive neuroscience, remote communication, digital technology, digital media

## Abstract

Human interactions are increasingly taking place from a distance through methods of remote interpersonal communication like video chatting and social media. While remote interpersonal communication has existed for millennia—with the first postal system arising in ∼2400 B.C.—accelerated advances in technology and the recent global COVID-19 pandemic have led to a dramatic increase in remote interpersonal communication use in daily life. Remote interpersonal communication presents a challenge to the field of social-cognitive neuroscience, as researchers seek to understand the implications of various types of remote interpersonal communication for the “social brain.” The present paper reviews our current understanding of the social-cognitive neural network and summarizes critical differences between the neural correlates of social cognition in remote vs. face-to-face interactions. In particular, empirical and theoretical work is reviewed that highlight disparities in the neural mechanisms of social perception, evaluation of social stimuli, human motivation, evaluation of social reward, and theory of mind. Potential impacts of remote interpersonal communication on the development of the brain’s social-cognitive network are also discussed. Finally, this review closes with future directions for research on social-cognitive neuroscience in our digital technology-connected world and outlines a neural model for social cognition in the context of remote interpersonal communication. For the field of social-cognitive neuroscience to advance alongside of the ever-evolving society, it is crucial for researchers to acknowledge the implications and concepts suggested for future research in this review.

## 1. Introduction

**FIGURE 1:**
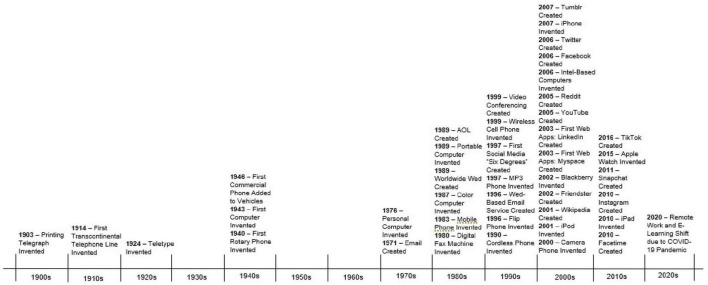
A timeline containing prominent developments in remote interpersonal communication in the past millennium. This timeline’s purpose is to exhibit the rapid progression and evolution in technology in recent years, especially following the invention of the World Wide Web (see Supplementary Appendix A for citations).

In Harlow (1959) seminal experiment, young rhesus monkeys preferred a “mother” made of cloth vs. one made of metal wire. In turn, those monkeys who were isolated to only experiencing a wire “mother” suffered great attachment-related consequences (Harlow, 1959). In present day, as technology seeps into every facet of life, we must wonder if our technological devices are the modern “wire mother” and question the implications of digitally mediated human communication for the brain’s social-cognitive network. Addressing these questions is critical for advancing the field of modern social-cognitive neuroscience. As depicted in Figure 1, there has been a dramatic increase in digitally mediated social communication and screen use in the past century. Despite this cultural shift, there is much still unknown about the impact of remote interpersonal communication on the social brain and this area of study should be prioritized in future cognitive neuroscience studies. The present review will examine these ideas in the context of the current literature.

**FIGURE 2:**
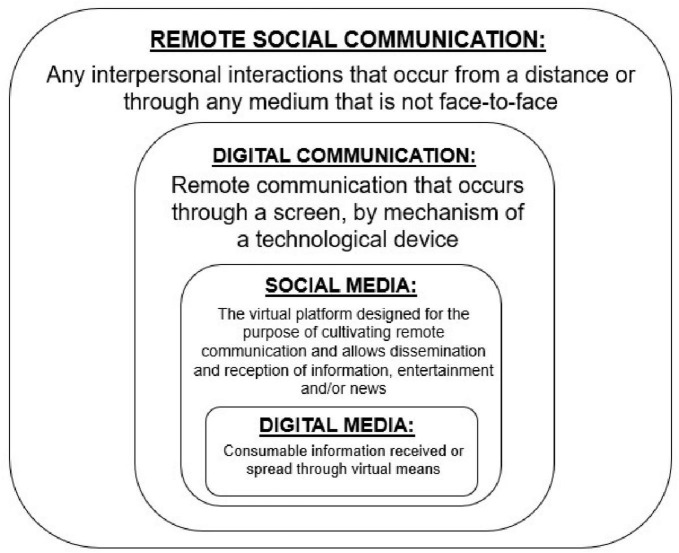
A visual representation of the keywords used in this review to describe the different types of remote communication.

Before going forward, however, we will provide functional definitions for key concepts that are covered in this review. We use *remote interpersonal communication* as the umbrella term for any interpersonal interactions that occur from a distance, i.e., when social communication is not face-to-face via physical proximity. In the present paper, we use the concise term of *digital communication* to represent any form of remote communication that occurs through a screen, in which the social partner is visually observable. *Social media* is the virtual platform designed for the purpose of cultivating remote interpersonal communication, but also acts as a mechanism for both receiving and disseminating information, entertainment, or news (Nguyen, 2021). *Digital Media* refers to consumable information received through virtual means and can either be the input or output of compatible forms of remote interpersonal communication (see Figure 2). All of these advances have been developed in the service of supporting human interaction and communication, but social cognition involving technology-mediated human communication is likely to be processed differently than in-person communication.

**FIGURE 3:**
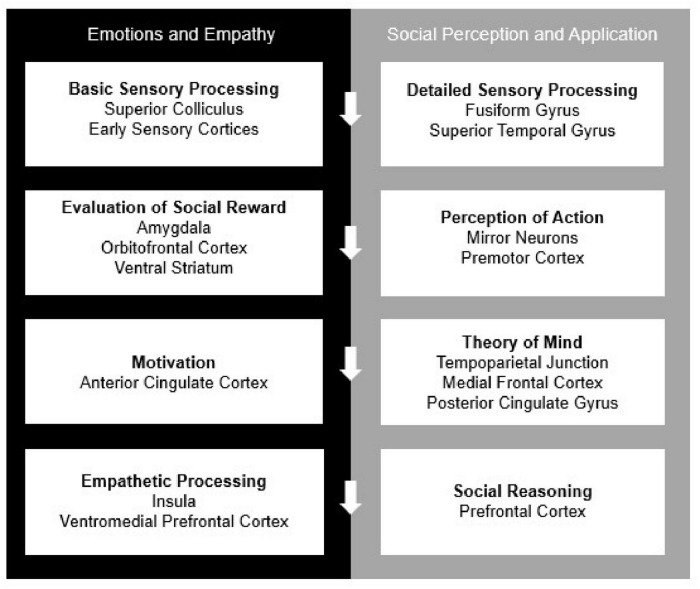
The current model of “The Social Brain,” adapted by Adolphs (2009). All processes work both independently and in concordance with one another to complete self-regulation, reappraisal, and contextual application.

Humans are social beings and in order to survive and thrive, we require neural systems that can support social behaviors. Social-cognitive neuroscience, as a well-established subfield of cognitive neuroscience, has described these neural systems through several, mostly overlapping, neural models of social cognition (Lieberman, 2007; Adolphs, 2009; Rilling and Sanfey, 2011). Among the most dominant and frequently cited models is that of Adolphs (2009), which is summarized in the following major section and depicted in Figure 3. The model describes neurocognitive mechanisms for processing and evaluating social stimuli and modulating these processes through application of context and regulatory mechanisms (Adolphs, 2009). Within this system are more specialized networks that focus on directing attention to relevant stimuli, regulating emotions, and sparking motivation for goal-directed behavior to ultimately receive positive social feedback (Brown and Brüne, 2012). In face-to-face interactions, these processes frequently involve making inferences and predictions of others’ behavior (Adolphs, 2010) from physical cues like tone of voice, body language and facial expressions (Britton et al., 2006). Consistent with Adolph’s model structure, social-cognitive neuroscience to-date has tended to highlight the specialized processes that occur before, during and after social behaviors. Additionally, there is a consensus in the field based on neurological evidence that functioning in social settings requires higher-order mechanisms in comparison to other non-social operations (Adolphs, 2010). However, a current challenge in the field is to understand whether and how remote interpersonal human communication impacts neural mechanisms of social cognition.

**FIGURE 4:**
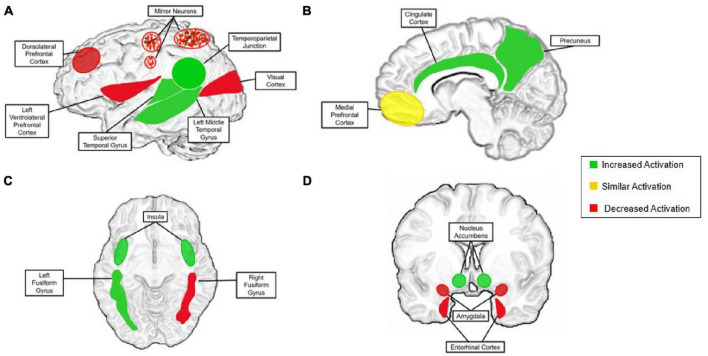
The proposed neural model for the remote social brain. “Increased activation” is activation that is present during aspects of remote communication more than during face-to-face communication (green). “Similar activation” is activation present at a similar level during remote communication and in-person communication (yellow). “Decreased activation” is activation that is substantially less present or completely absent in remote communication compared with that of face-to-face (red). **(A)** Left lateral view of the brain; speckles are to indicate mirror neurons, in that they are affected, but the region as a whole is not; **(B)** sagittal view; **(C)** axial view; **(D)** coronal view. Superior colliculus (decreased) and is not pictured for figure clarity.

While social cognition is relatively distinct, research supports that its neural correlates derive from basic pathways of non-social processes (Brown and Brüne, 2012). There are vast connections between social and non-social systems, but there is speculation that remote interpersonal communication does not fit comfortably into either of the afore-mentioned categories. In recent years, digital technologies have become increasingly capable of simulating in-person interactions without being face-to-face such as platforms for video chatting. This new phenomenon may result in functional brain activation patterns that cannot be classified as “social cognition” or “non-social cognition” under current neural models. To consider this question, we will first summarize current findings on neural mechanism of social cognition and synergize existing evidence to consider how these understandings either can or cannot be applied to remote interpersonal communication. With this foundation, we then propose how neural mechanisms that support social cognition during remote interpersonal communication may differ from those involved in face-to-face interactions—particularly social-cognitive mechanisms of motivation, social connectedness, and reward processing. These discrepancies are depicted in Figure 4, described throughout the manuscript and summarized in the discussion.

## 2. Sensory processing of social stimuli

Humans possess unique and complex cognitive abilities to process social stimuli. Adolphs’s (2009) model of social cognition also describes the important role of the superior colliculus in subconscious visual processing specifically in directing eye movements to social stimuli. Additionally, the model describes how the early sensory cortices are associated with deferring perceptual information to more complex socially tuned processing areas. Beyond these foundational structures, other brain regions are more specialized and complete higher-order functioning in the context of sensory processing. In particular, the fusiform gyrus is a highly specialized brain region for recognizing and processing faces, even in non-social stimuli (e.g., face-like patterns; Britton et al., 2006). This allows for the visual perception of facial expressions in-person, but also through digital media like pictures and videos. The fusiform gyrus is crucial for interpersonal communication, and may also function similarly when social partners are not physically present. Indeed, neuroimaging research on social media use have found prominent activation in the left, but not right, fusiform gyrus during the use of social networking site “Facebook” (Turel et al., 2017). Notably, the left fusiform gyrus has been reported to respond to face-like stimuli, while the right side’s function is associated with decision making involving face-like stimuli (Meng et al., 2012). The superior temporal gyrus functions to recognize and interpret emotional social stimuli, such as body language, gesture, the movement of mouths and eye gaze (Britton et al., 2006; Adolphs, 2009). However, this structure appears to have a different function within the context of remote interpersonal communication, which will be discussed below.

### 2.1. Sensory processing of social stimuli in remote interpersonal communication

Several brain structures that are acknowledged in the mechanism of sensory processing have similar functions in remote social stimuli. Notably, research on the superior colliculus is typically performed through methods involving the observer having to view movements on a digital screen (Cutsuridis et al., 2014), or in earlier studies through a cathode tube ray monitor (Horwitz and Newsome, 1999). Although these sensory structures exhibit some activation during different experiences of remote interpersonal communication, we hypothesize that the activation in these regions will not be as prominent as that of in-person social interaction. Neuroimaging studies have provided evidence that the processes involved in fulfilling sensory perception are incomplete when interacting through digital communication (Derks et al., 2008). Therefore, we suggest continued investigation into the richness of sensory experiences through remote interpersonal communication.

Notably, prior research on the neural basis of social stimuli perception typically relies on digital media to present visual and auditory social stimuli. For example, faces are perceived from projected images or voices from headphones in a magnetic resonance imaging (MRI) scanner (Hove et al., 2013). Thus, our understanding of basic social stimuli processing mechanisms, particularly in the visual and auditory domains, should directly represent social stimuli processing via remote interpersonal communication. Other sensory modalities involved in social interactions (e.g., somatosensory, olfactory) are less frequently studied (Adolphs, 2009) and less likely to be involved in social communication and interactions through current digital technology. However, current and future technologies that target these sensory modalities (e.g., haptic suits) are likely to engage primary sensory and association cortices in their associated systems, as we have observed in visual and auditory systems. In addition to these suggestions, researchers can use brain imaging techniques that allow participants to interact face-to-face. Many neuroimaging studies on social interaction utilize functional MRI, but this prohibits genuine face-to-face social interactions. Techniques such as electroencephalogram (EEG) or functional near-infrared spectroscopy (fNIRS) allow researchers to record brain activity during in-person interactions instead of viewing social stimuli on a screen.

### 2.2. Representing actions

Mirror neurons activate during the observation of a social partner, with findings indicating a role in processing and learning action-outcome associations from another’s behavior (Brown and Brüne, 2012), relating to reinforcement learning (Adolphs, 2010). Such observational learning is thought to form a foundation for learning social norms, as the observer comes to implicitly understand which behaviors are acceptable and when (Brown and Brüne, 2012). The firing of mirror neurons in accordance with continuous reinforcement learning allows for the development of higher order abilities, such as being able to evaluate social feedback (Adolphs, 2010). Foundational research, particularly with non-human primates, found strong support for mirror neurons in the ventral premotor cortex and inferior parietal lobule (Gallese et al., 1996; Keysers et al., 2003; Fogassi et al., 2005). Since then, cognitive neuroscience has largely abandoned the view that mirror neurons are isolated to these select motor regions (Mukamel et al., 2010). Indeed, evidence of mirror neurons has been observed in cortical midline structures (medial prefrontal cortex, anterior cingulate cortex, and precuneus) during integration in systems of theory of mind and empathy (Uddin et al., 2007; Carrillo et al., 2019). Such findings suggest that mirror neurons support learning through direct observation and mental simulation (Saito et al., 2018).

#### 2.2.1. Representing actions in remote communication

There is presently a dearth of research that directly compares neural mechanisms of social stimuli evaluation across face-to-face and digital remote contexts. In developing expectations, we reflect that digital communication can include screens to simulate or approximate real-time, face-to-face communication. On the other end of the spectrum, other forms of remote interpersonal communication such as phone calls provide no visual non-verbal cues. In this way, technology like video-mediated social communication is an intermediary between social and non-social contexts and, more broadly, different types of remote interpersonal communication should differentially engage social and non-social neurocognitive mechanisms.

As of recently, researchers have begun to focus on deficits in mirror neuron firing from observing human actions through digital communication vs. in-person observation. For example, research using infants show greater sensorimotor activation in response to live observation of an actor manipulating an object compared with observing an object moving on its own (“ghost condition”), but there is no activation difference between these conditions if they are shown on a video recording (Shimada and Hiraki, 2006). Such findings suggest that observing someone through video, even during live calls, is less likely to evoke a mirror neuron system response (Dickerson et al., 2017). When completing a video call, individuals receive significantly less non-verbal cues, making it more difficult to accurately perceive the communication and feel socially connected (Gronewold and Engels, 2022). These findings hold implications for a number of digitally mediated communication contexts, including remote schooling, work, and socializing (e.g., video chat). Further, digitally mediated social communication appears to require greater neural resources than face-to-face communication—potentially triggering an increased need for integration across sensory modalities and cognitive networks (Dickerson et al., 2017). Further investigations are vital to understanding the perception of others through digital media, especially with added issues like low-resolution video quality (Derks et al., 2008; Dickerson et al., 2017).

First, with both the complexity and significance of mirror neurons, it is important to consider how this system is affected through remote interpersonal communication. Research indicates that when viewing digital media, mirror neurons are still activated to some degree, but completion of firing is frequently not reached due to visual restrictions resulting from cropped screens (Derks et al., 2008). Additionally, with the multitude of accessible content, humans are more likely to consume media that aligns with their established interests or viewpoints, limiting their intake of novel information (Keysers and Gazzola, 2010). Such findings are particularly important to consider as the accessibility of media allows for the consumption of harmful, explicit, and offensive materials—including acts of extreme violence. This is a rather new concept as the digital age continues to evolve, and both short-term and long-term effects of this digital media consumption are not fully understood and must be addressed by social neuroscientists (Hargrave and Livingstone, 2009). Mirror neurons fire during the viewing of this content, and that could be detrimental to the social reinforcement learning of individuals, especially young children (Keysers and Gazzola, 2010). We may begin to see a shift in social norm related behaviors from the younger generations as they learn how to act from those they are observing through digital content, opposed to that of in-person interactions.

Through most instances of reinforcement learning and mentalization, mirror neurons fire and allow humans to learn through observation or prediction of social feedback (Brown and Brüne, 2012). Due to the difference in social norms in the online world compared those in-person, individuals may begin learning different norms which can ultimately affect their in-person behaviors. Firstly, digital communication does not always require an individual to wait their turn to speak, a prominent norm in face-to-face interaction (Meshi et al., 2015). Activities like texting and commenting, an individual has the opportunity to send continuous messages without even acknowledging other users. Digital communication also allows longer periods of time before responding to communication compared with face-to-face communication (Meshi et al., 2015). These behavioral patterns, in conjunction with greater self-disclosure and lack of need for politeness, may have negative impacts on face-to-face communication where such actions violate social norms (Meshi et al., 2015). It is understood that in most cases, humans have control over their behavior in accordance with the social cues available from the context (Adolphs, 2010). However, repeated negative reinforcement to social norm violations in online communication can be detrimental to developing and implementing appropriate face-to-face social actions. That said, actions, words and behaviors that would result in the experience of negative social feedback in-person may not cause the same effect in online environments due to differences in virtual etiquette. It is critical for more social-cognitive reinforcement learning studies to take place while monitoring mirror neurons and other mechanisms that govern these processes to understand how they differ through digital communication.

## 3. Social reward

Social reward is a broad term for the receipt of positive social feedback or social satisfaction. Although many structures and networks both process and evaluate potential rewards, key structures include the amygdala and the frontostriatal reward network (particularly the ventromedial prefrontal cortex, ventral striatum; O’Doherty, 2004). These structures process a multitude of rewards from social experiences including positive social connection, subjective feeling of winning, and pleasure (Borland et al., 2018). These structures are likewise involved in neural signaling for negative social experiences (or absence of reward) including loss, withdrawal, separation distress, and loneliness.

The function of the reward network is to support incentive-based learning and adaptive, goal-directed behavior (Haber and Knutson, 2010). Neuroimaging research has yielded information regarding the context-dependent nature of the reward network, which appears to respond differently during social and non-social cognition (Brown and Brüne, 2012). This complex system can also modulate social reward values (subjective utility) based on internal states (e.g., motivation; Brown and Brüne, 2012). Particularly, the ventromedial prefrontal cortex activates when one is observing and evaluating the reward-related outcome received by another individual (Brown and Brüne, 2012). This observational learning increases motivation for reward-seeking behavior upon receiving positive social feedback (He et al., 2017). Cognitive neuroscientists are urging a new focus of research of this system’s implications with social and digital media (Meshi et al., 2015). In particular, the reward network, along with the amygdala, shows substantially altered functioning in drug addiction (Rogers et al., 1999). These same neural mechanisms underlie dysfunctional seeking of positive feedback on social media (He et al., 2017). In particular, it processes cues from environmental stimuli to avoid negative feedback or experiences (He et al., 2017). The amygdala is heavily linked with social reinforcement centers in the brain as it functions by integrating external stimuli to previously learned behavior, ultimately prompting goal-directed behavior for a positive outcome (He et al., 2017).

With respect to mechanisms of social connectedness specifically, opioids and oxytocin appear to play key roles. When released in the brain, oxytocin has an inhibitory effect on general feelings of distress (Panksepp, 2014) and has been observed to assign valence cues to what is observed in social situations (Borland et al., 2018). It is a prominent neurochemical that facilitates social attachment (Rilling and Sanfey, 2011), similarly to that of opiates. The opiate neurochemical plays a tremendous role in positive social connection related processes that researchers have suggested an opioid theory of attachment. The opioid theory of social attachment began with evidence that individuals who were medically treated with opioids reported lower levels of loneliness but were significantly more sensitive to unfavorable social feedback (Panksepp, 2014). Later research indicated that the opiate system in the brain is activated during periods of social connectedness, feelings of love, and experiences of positive attachments (Panksepp, 2014). This system appears to regulate social attachment through opioid-mediated separation distress (Panksepp, 2014).

The opposite of social connection is social rejection. Social rejection is classified by the lack of feeling of belonging in a social group, or in society as a whole. Although categorized as an emotional phenomenon, experiencing social rejection activates the dorsal interior insula and the secondary somatosensory cortex, both of which are involved with physical pain (Kross et al., 2011). To avoid this negative state, humans are motivated to make connections with others, and more importantly, focus on avoiding social rejection. When brain regions governing emotional and anxiety regulation are defective, individuals appear to have a lower threshold for tolerance of ostracization (Bach et al., 2019). It is possible that consistent engagement with social media generally increases the likelihood of rejection experiences (Andrews et al., 2022). However, conflicting viewpoints claim that digital communications allow individuals to feel more connected to others (Savci and Aysan, 2017).

### 3.1. Social rewards in social media

Current research suggests that social media increases social orientation toward quantifiable social rewards that are unique to social media (Meshi et al., 2015). That is, the number of followers, likes, and comments an individual receives is plainly visible for both the receiver and those in the social media network (Meshi et al., 2015). Such explicit quantitative measures of social popularity exist in non-digital social settings (e.g., school children clustering in social groups of different sizes) but are less embedded as a central feature of in-person social interactions. The core feature of popularity quantification in social media has been proposed to trigger increased attention to the size of social networks, with a decreased emphasis on making deep connections (Wagner, 2015). Further, extant research suggests that the explicit quantification of social rewards in social media may impact the amygdala and reward network in a similar way to drugs of abuse, resulting in addictions to social media. Indeed, recent studies have shown that there is a negative association between reported feelings of social connectedness and social media addictions and dependencies (Savci and Aysan, 2017), with young people at highest risk (Andreassen et al., 2017). There is current controversy over whether the malignant outcomes of spending too much time on technology should be considered a use disorder (He et al., 2017). Here, we argue that social media dependency should be associated with other behavioral and chemical addictions, due to similarities in biological factors, and behavioral and emotional symptoms.

Social media and technology dependencies elicit behavioral symptoms comparable to that of established addictions such as withdrawal, tolerance, and continued use after daily functioning is impacted negatively (Savci and Aysan, 2017). The outcomes of these addictions can be detrimental, such as that of intentional isolation, depression, low self-confidence, lack of inhibitory control, decreased performance in work or school, and quality of sleep (He et al., 2017; Savci and Aysan, 2017). These psychological and behavioral outcomes may be due to structural changes in the brain regions that support response to and evaluation of social rewards. Most notably, recent neuroimaging research has revealed a negative association between social media use and bilateral amygdala volumes—similar to that observed in additions to gambling or drugs of abuse (c). Lower volumes of gray matter in the amygdala may promote continued and compulsive use of technology, with a constant chase to keep up with increasing tolerance (He et al., 2017). Critically, however, where reduced gray matter volume in the cingulate cortex is typical in other addiction disorders, level of social media addiction was associated with larger volumes in the anterior/mid-cingulate cortex (He et al., 2017). This discrepancy may further the debate on classifying overuse of technology as an addiction and underscores the need for additional research on this topic.

## 4. Social rewards: social connection and rejection in remote communication

It is currently unknown whether needs for social connectedness can be satisfied through online communication. On one hand, findings suggest that increased use of technology prevents occurrences of face-to-face interactions and can ultimately lead to great feelings of loneliness (Savci and Aysan, 2017). On the other hand, it is contested that the access to online communication actually facilitates greater sociability, in that more connections can be easily made (Savci and Aysan, 2017). Furthermore, ongoing research has demonstrated positive outcomes of social media use. Firstly, remote interpersonal communication makes it easier to maintain social relationships by bridging physical distance (Savci and Aysan, 2017). And as a challenge to the hypothesis that technology-based social interactions discourage in-person interactions and increase loneliness, self-reported social media use was associated with reduced feelings of loneliness and greater sense of social connectedness (Savci and Aysan, 2017). It is inferred that this is because having communication at the palm of one’s hand allows people to feel constantly connected to others (Deters and Mehl, 2013). It has been proposed that social media meets certain social needs, but not all (Grieve et al., 2013). Differences in the ability to address social needs may be due to discrepancies in the neural processes that support social communication in remote and face-to-face contexts.

A more positive view comes from structural neuroimaging research, which has found similar relationships between amygdala volumes and online, as well as “real world” social network sizes (Kanai et al., 2011). Notably, the same study found that online social network size—but not real-world social network size, predicted gray matter volumes in the right superior temporal sulcus, left middle temporal gyrus and entorhinal cortex. Given these regions’ roles in social perception associative memory, such results suggest that developing and maintaining large remote social networks requires greater social-cognitive capacities.

The opioid theory of attachment is also relevant in the realm of social connectedness. The brain’s endogenous opiate system releases neurochemicals that both promote pleasure and relieve pain (Watkins and Mayer, 1982), similarly to when individuals feel socially satisfied (Panksepp, 2014). People with opioid addictions continue ingesting the drugs to reach this level of pleasure, comparable to those with social media dependencies who compulsively use technologies. Researchers have found direct associations in opioid addictions and social connectedness between drug dependence and meaningful social relationships, the race to keep up with tolerance and the drive to avoid loneliness, and with drug withdrawal and anxiety from exclusion (Panksepp, 2014; see Table 1). These significant overlaps should urge researchers to integrate the opiate theory while conducting research on social media and technology addictions.

**TABLE 1:** Demonstrates the parallels in the brain between opioid addiction and social connectedness (Panksepp, 2014).

Opioid addiction	Social connectedness
Drug dependence	Need for meaningful social relationships
Race to keep up with tolerance	Motivation to maintain connections
Drug withdrawal	Anxiety from social exclusion

When social connectedness is absent, separation distress is felt, as both operations stem from the same neural system (Panksepp, 2014). Feelings of loneliness and general lack of social connection can stem from a multitude of contexts, but prominently through social rejection. There is an abundance of evidence that repeated social rejection is associated with lower gray matter volume, particularly in regions mediating feelings of social anxiety and rejection sensitivity. Recent studies have investigated gray matter volume differences during periods of positive and negative social experiences and have found reductions during times of social exclusion (Bach et al., 2019). Lower gray matter volumes in the posterior temporal sulcus have been associated with greater feelings of loneliness; however, it is unclear whether lower gray matter preceded or followed feelings of loneliness (Quadt et al., 2020). Reduced gray matter in the posterior temporal sulcus has also been associated with lower social skills. Again, however, additional research is required to determine a causal chain between (Quadt et al., 2020).

Both the insula and inferior frontal gyri have apparent gray matter reductions during the experience of such (Bach et al., 2019). More specifically, research has indicated a social rejection system including involving the insula, left anterior cingulate cortex, and the inferior frontal cortex (Bach et al., 2019). In investigating the pathways related to these regions, the discovery of a network controlling a downward regulation of social affliction was found in connection with the ventral striatum (Bach et al., 2019). Lower volumes of gray matter along this region are thought to represent chronic social anxiety but have the potential to increase motivation in other modulatory systems to instigate social interactions to decrease this feeling (Bach et al., 2019). It is crucial to continue research on this system, as exploratory actions have not been subsequently repeated. Furthermore, it is not yet understood whether social anxiety is a result or promoter of the deficits in gray matter along this network.

## 5. Higher-level social cognition processes

### 5.1. Motivation

Humans have compelling and natural biological drives to meet and maintain homeostasis in terms of emotions, comfort and satisfaction through social reward (Britton et al., 2006). These requirements all instigate social interactions in order to meet an adequate level of contentment. Additionally, humans have intrinsic motivation to avoid aversive outcomes or negative emotions, including those arising from social interactions (Britton et al., 2006). A major proponent of satisfying motivations is the anterior cingulate cortex. As discussed, it has a primary function of avoiding negative social feedback (He et al., 2017). Within the Adolphs model of social cognition, the anterior cingulate cortex is a highly integrated system that promotes motivation through collaboration with emotional mechanisms and sensory perception networks that provide information about the context of the environment (Adolphs, 2009). In the brain, the insula exhibits high levels of activation during the satisfaction and/or lack of these biological needs (Britton et al., 2006). During such experiences, the insula supports visceral processing and negative emotions, such as disgust with more complex pathways insula involve socio-emotional functioning (Uddin et al., 2017). The process of reaching the preferred outcome through the seed of motivation utilizes highly complex, integrated systems involving neighboring structures including the tempo-parietal junction and medial prefrontal cortex (Adolphs, 2010). Furthermore, the amygdala plays a key role in perception and evaluation of threat, which motivates the avoidance of actions with aversive outcomes and is highly tuned to social stimuli (Adolphs, 2003).

### 5.2. Cognitive control

In order to accomplish the anticipated outcome of motivation, one must be able to evaluate the current social context, and exhibit goal-directed behavior in accordance with it. Cognitive control is an advanced social ability that involves developing planned actions in social settings (Adolphs, 2010). This function allows individuals to navigate their social environments by evaluating the present situation, determining appropriate behavior, and ultimately carrying out optimal actions. The cognitive control pathway includes the dorsolateral prefrontal cortex, inferior frontal gyrus and precuneus (Yang et al., 2021). This system derives from basic visual perception pathways but extends beyond simple mechanisms to be utilized in decisions of social behavior (Herd et al., 2006), especially in contexts where positive social feedback is expected (Yang et al., 2021). Cognitive control is necessary for proper social functioning, and disruptions in this mechanism lead to inability to detect social deception (Adolphs, 2010) and causes deficits in emotional regulation (Ochsner and Gross, 2005). Cognitive control is understood to be highly tuned to social stimuli, with prominent connections to the ventral attention network and the frontoparietal control network (Wong et al., 2022). The ventral attention network recruits the inferior parietal lobule and tempo-parietal junction to regulate emotions and make decisions about where to direct attention (Viviani, 2013). Subsequently, the frontoparietal control network promotes goal directed behavior, but is thought to be highly domain specific (Spreng et al., 2010). Researchers note that it is important to understand what is occurring in terms of cognitive control when these networks exhibit reduced activation (Viviani, 2013), and it is unclear whether cognitive control manifests itself the same way in non-social communication or remote interpersonal communication (Meshi et al., 2015).

### 5.3. Mentalizing the self and others

Another intricate human component of social cognition is the ability to internally process abstract concepts of others, known as theory of mind (Lieberman, 2007). This sophisticated mechanism involves mentalizing, or thinking about the mental states of others (Keysers and Gazzola, 2007). At the crux of theory of mind is the insula, which is activated during introspection and reflection, and exhibits prominent connections to midline structures for high level processing of these states (Keysers and Gazzola, 2007). The insula is stimulated during periods of empathy, which is both a major aspect of theory of mind and is also unique to humans (Britton et al., 2006). This is an introspective mechanism that does not require direct observation of a social or non-social stimulus. In social decision making, the insula is involved in expectations of negative social feedback (Yang et al., 2021). Another prominent region that is heavily involved in theory of mind is the tempo-parietal junction (Adolphs, 2010) which has the role of adding context to social behaviors in order to reach the goal of mentalization (Carter and Huettel, 2013). Similarly, the medial prefrontal cortex plays a role in the mentalization of both intrapersonal and interpersonal states (Britton et al., 2006). The amygdala contributes to evaluating the social emotions of the self and others (Britton et al., 2006), as well as speculating on the intentions of others’ actions through threat recognition (Adolphs, 2003). These structures and their basic functions provide higher-order abilities that appear unique and specialized to social environments.

### 5.4. Abstract social cognitive processes in the digital age

In remote interpersonal communication, theory of mind is perhaps the most heavily recruited neural process. It is required to determine and navigate the social and emotional states of others, but during digital communication, physical, the non-verbal cues that are typically present in face-to-face interaction are digitally mediated or absent. Recent work indicates that, similar to in-person interaction, theory of mind in online contexts invokes activation in areas like the dorsomedial prefrontal cortex and the tempo-parietal junction (Quadt et al., 2020). The superior temporal gyrus has been observed to be heavily recruited in virtual instances of theory of mind, and increased volume in this region appears to be associated with frequent social media use (Turel et al., 2017). In contrast, there appears to be a notable disparity during experience of theory of mind in the virtual domain during reputation regulation exhibited by reduced activations in the dorsolateral prefrontal cortex (Adolphs, 2010). This activation pattern is typically not seen in conventional, real-world experiences of theory of mind (Adolphs, 2010). Theory of mind processing may also show divergent neural mechanisms for in-person vs. remote social interactions if social feedback signals are salient. As described above, digital contexts may increase the focus on one’s social group or audience, prompting one to engage theory of mind to execute goal-directed behavior (Meshi et al., 2015). The mentalization of receiving an acceptable outcome from calculated behavior is modulated by the dorsolateral prefrontal cortex which puts emphasis on obtaining quantifiable social feedback. This desirable outcome is processed in the brain through the reward network (Gerlach et al., 2014).

Additionally, the use of social media recruits the complex process of self-referential thought, which prompts individuals to create content that will directly benefit them in their online reputation. This process recruits regions such as the medial prefrontal cortex and posterior cingulate cortex, both of which are responsible for interpreting in-person social feedback (Meshi et al., 2015). Neuroimaging studies that monitored brain activity when individuals received an abundance of “likes” on social media exhibited notable activations in the mentalizing system, specifically in the precuneus and tempo-parietal junction (Sherman et al., 2018). The near constant consumption of digital media poses a question as to whether online social interactions are enough to meet intrinsic social needs of having satisfactory interpersonal connections. We have established that online relationships often lack the richness and depth of true social interactions, and that the neural processes of perception in these settings do not directly match. The connections and discrepancies of social media use to real interactions implies that the neural mechanisms are similar, but different (Derks et al., 2008).

### 5.5. A developmental approach to recent influx of remote interpersonal communication

When the COVID-19 Pandemic struck in March of 2020, the lives of nearly every single human being on the planet were impacted. This event perhaps multiplied the use of remote interpersonal communication as it was the only possible way to continue daily life during periods of mandatory social isolation. Those who have once experienced “normal life” in social settings, for the most part, were able to assimilate to these changes. However, it is critical to understand both the impact and long-term effects of increased remote interpersonal communication use on young people. Based on animal models of social isolation, these young people who were isolated from an early age or even from birth may suffer irreversible cognitive and motivational deficits. We will speculate on these potential issues and urge researchers to perform longitudinal research on these young individuals based on both social isolation, and increased use of remote interpersonal communication, which has skyrocketed in this population. We currently do not know the long-term cognitive consequences of technology and digital media overuse in young people.

To understand the effects of social isolation at a young age, we must consider the implications of it that are currently understood in the literature. Social isolation can be detrimental to individuals as its impact on neural mechanisms are similar to that of physical pain (Kross et al., 2011). As we previously discussed, chronic overuse of social and digital media can result in physical social isolation, and again, it is speculated that digital means of communication are not sufficient to satisfy the intrinsic needs of connectedness. Social isolation is a step beyond social rejection and involves different brain networking that can have harmful consequences, such as depressive symptoms (Panksepp, 2014). This phenomenon stems from overactivation of the pituitary adrenal region, creating a lower tolerance for and higher withdrawal from needs of connection (Panksepp, 2014). We discussed that the highest risk group for social media addiction is adolescence, which also is known to be a time of critical period for social development (Teicher et al., 2004). Children learn through observation and reinforcement, and removal from situations involving social interaction can completely prevent this process (Santrock, 2023). This critical period of social development is associated with rapid brain growth and structure maturation, and any disruption to this can have lasting negative consequences (Santrock, 2023). With more and more young people participating in the near constant use of social media, and subsequent potential social isolation from peers, the implications of this issue is critical to understand in modern times.

### 5.6. The effect of social isolation on brain development

Again, to understand the potential cognitive deficits of socially isolated young people who also are more inclined to use digital communication, we must consider the neural development that takes place during this time. Myelin is both prominent and crucial for proper brain development. It is a plentiful lipid in the brain that surrounds fibers and promotes the speed of neuronal activity (Boggs, 2006; Hartline, 2008). Myelin is highly flexible in that it can be regenerated both easily and quickly after degeneration, commonly caused by negative stimuli from the environment (Lehmann et al., 2017). Studies have shown a direct association between the downregulation of myelin and chronic social defeat (Lehmann et al., 2017). Additionally, repeated stress reduced the continuation of myelin and results in shorter lengths of the protein (Lehmann et al., 2017). With its observed plasticity, myelin can recover from these negative effects in adulthood (Lehmann et al., 2017), but unfortunately, during critical periods of development in younger people, myelin production can be perpetually stunted and exhibit reduced flexibility. Infrequent social interaction in childhood and adolescence was observed to prevent full maturation of myelin fibers, an irreversible phenomenon when experienced early in life (Makinodan et al., 2012).

Animal models of social isolation have also exhibited that when isolated for as little as 2 weeks, the interruption in myelin growth significantly altered development of the prefrontal cortex, and normal functioning was not recovered when introduced to social stimuli (Makinodan et al., 2012). Studies performed on mice who were socially isolated from a young age found that no form of therapy later in life was able to significantly solve the biological deficits caused from solitude (Panksepp, 2014). This differs from similar research on adult mice, who were able to recover from social isolation, plausibly because they had normal myelin development during childhood (Lehmann et al., 2017).

Myelination is also associated with brain processing speed, and individuals with disruption in development of it have reduced cognitive abilities that affect more than social intelligence (Santrock, 2023). Overall, a decrease in brain function and volume has been observed in adulthood when individuals were socially isolated in childhood (Santrock, 2023). Myelin promotes working memory and learning by electrical impulses derived from plasticity upon receiving different types of social feedback (Fields, 2008). Research has shown that the disruption of myelin development can have consequences when experienced beyond the critical period, suggesting that interruptions up to or around age 20 have consequences (Fields, 2008). Incomplete myelination specifically in the prefrontal cortex show signs of impaired decision-making, which if absent, can be very harmful to an individuals’ self-control and functioning in society (Makinodan et al., 2012). These significant discoveries need to be examined in the modern age, through children who are socially isolated due to both digital forms of communication and the state of the world through the COVID-19 pandemic.

## 6. Implications due to the COVID-19 pandemic

The COVID-19 pandemic changed the course of society across the entire world, especially that of young children. Over 1.5 billion children moved into lockdown, were removed from in-person schooling and experienced social interactions limited to their families (Montag and Elhai, 2020). Teachers had to adjust their curriculum to a virtual format, which resulted in a very different learning mechanism, especially for those who were in school for the first time. These students had to miss out on valuable social experiences like play, reinforcement learning, exploration, and making connections with peers (Montag and Elhai, 2020; Steed and Leech, 2021). In early childhood education, most instruction is completed through play-based learning, which is not fully possible to do over a virtual format (Garbe et al., 2020). This population is also most susceptible to technology overuse (Montag and Elhai, 2020), and the introduction of digital media to every facet of their lives creates new challenges that have yet to be fully understood by researchers. Studies must be performed on both the direct and indirect effects of excessive technology use and lack of social interactions on young children during their critical period of social development (Montag and Elhai, 2020).

If an individual never gets the experience of connecting with their peers during the largest period of brain growth, there can be detrimental effects. In addition to deficits in social intelligence and memory, researchers have found that this can also result in problems navigating through and expressing emotions (Panksepp, 2014). Also, children who do not have access to developing healthy social relationships suffer consequences later in life like increased social distress, anxiety, codependency and development of psychiatric illnesses (Panksepp, 2014). Brain mechanisms of social isolation overlap heavily with that of neglect, expressed through declined volumes in the corpus callosum (Teicher et al., 2004). Neglect was also unfortunately common during the COVID-19 lockdown, as parents reported increased stress when having to work from home and had difficulties transitioning into being both the guardian and teacher (Garbe et al., 2020). In order to keep children occupied during work-from-home situations, parents often turned to allowing increased screen time for their children (Garbe et al., 2020). With this, children started being accustomed to using digital technology for school, play, entertainment, and social interaction. Since this issue arose in very recent years, the long-term effects of this are both under-researched and unknown.

This critical period for development is a large umbrella that also encompasses periods of establishing a sense of motivation and emotional regulation. The implications of the COVID-19 pandemic is both hard to explain to children, and difficult for them to understand, especially at the beginning in which there was world-wide uncertainty. This effect ultimately led to increased cases of immense anxiety in children, which ultimately affected every facet of their lives (Garbe et al., 2020). Frequent, negative emotional states in children lead to deficits of emotional regulation, motivation, learning and memory (Tortella et al., 2021). The brain must exert more effort in attempting to maintain a baseline of emotional homeostasis, which in turn, exhausts resources used for retaining information in learning environments (Tortella et al., 2021). Increased stress produces an overabundance of cortisol, which acts as an inhibitor for the acquisition of novel environmental stimuli (Tortella et al., 2021). Overall lack of motivation to attend online schooling was reported to occur during the COVID-19 lockdowns, all of which inhibit neurotrophic tendencies that promote brain development (Tortella et al., 2021). Although this mandate was out of the public’s control, it may produce lasting negative effects on children, and we urge that researchers focus on these issues and develop necessary interventions.

## 7. Impacts on adolescents

Although young people may be most heavily affected by social isolation and digital technology overuse, adolescence is also an important stage of life impacted by the same implications but in a different way. There appears to be a critical period in which repeated social rejection can permanently stunt the development of various brain regions, resulting in a lasting elevated level of rejection sensitivity. Neural circuitries including the amygdala are exhibited to regulate feelings of social rejection (Bukowski et al., 2018). If these networks are being constantly fed by exclusion, they exhibit various forms of down-regulation and are damaged rather than strengthened during a vital period of development (Bukowski et al., 2018). Similar investigations of this topic have focused on adolescents who have been diagnosed with anxiety and depression. Increased activations in the striatum and its subsequent connectivity patterns were observed during the experience of negative social feedback in these individuals (Bukowski et al., 2018). When researchers further explored this pattern of heightened activation, it was discovered that repeated instances of rejection create a feeling of desperate longing for healthy social interaction (Quadt et al., 2020). Interpretations of these findings indicate that individuals expressing these prominent chemical differences may be more susceptible to rejection sensitivity and ruminate more on negative social feedback (Quadt et al., 2020).

Adolescence is a critical period of social development, and there is current debate as to whether the continuous access to social media is beneficial or harmful in this age group. It appears that interpretations of findings tend to emphasize the negative effects. Although social media provides more opportunities to be connected with others, it can result in expanded opportunities for social rejection to occur. The period of adolescence is when rejection sensitivity appears to be at its peak and is also the age group most likely to participate in social media (Andrews et al., 2022). In various studies, this age group has been observed to spend significantly more time dwelling on negative social feedback, and digital media only creates more situations for that to occur (Andrews et al., 2022). As mentioned earlier, social media provides a publicly visible and quantifiable means for social networks. It is speculated that adolescents will take less engagement on social media posts as a form of negative social feedback which can have consequences matching that of in-person social rejection. As discussed, if individuals weigh their online social network as heavily as that of in real life, the negative consequences will be just as problematic (Lovnik, 2014).

### 7.1. Neural model of the remote social brain

As highlighted in this review, there are many discrepancies at the neural level when comparing brain structure and activations of remote contexts to in-person interactions. Although there is some overlap between contexts, there are a number of potential differences that require further investigation. We established that remote interpersonal interaction is neither a fully social nor non-social process and this topic needs to be explored further to determine its precise neural mechanisms. Through our synthesis of the current literature, we have created the neural model of remote interpersonal communication depicted in Figure 4. This model describes expected differences in the neural correlates of social cognition during remote interpersonal interaction compared with face-to-face interaction. First, perceptual activity in the brain is disrupted when viewing individuals through digital means, resulting in incomplete firing of mirror neurons in the premotor cortex. In terms of sensory perception, we see an overall decrease in key structures such as the superior colliculus and early sensory cortices when stimuli is presented via a virtual platforms. Notable differences in processing of social stimuli are seen in the fusiform gyrus, in which only the left side is activated when viewing faces through digital media.

Additionally, substantial differences in the processing of social reward are present when comparing in-person interactions to remote interpersonal communications. With the exception of notable activation in the posterior cingulate gyrus shared in both contexts, remote interpersonal interaction appears to activate other key structures of the social reward system to a higher degree than in-person interactions, possibly due to the instant gratification of quantifiable social feedback. Increased activation has been recorded in the nucleus accumbens, cingulate cortex, left middle temporal gyrus, and the right entorhinal cortex when receiving digitally delivered social rewards. We do, however, observe the greatest context differences are observed during abstract cognitive processes such as theory of mind. Current findings support that theory of mind mechanisms are more significantly recruited in remote interpersonal communication. This is due to partners not being face-to-face, ultimately requiring heavy mentalization that may not be present when communicators are physically observable. Related work has shown increased activation in structures supporting theory of mind functions, such as the tempo-parietal junction, medial prefrontal cortex, precuneus, dorsolateral prefrontal cortex and insula. Next, as the superior temporal gyrus underlies recognizing and interpreting physical emotional stimuli in face-to-face interactions, it tends to be recruited heavily in remote interpersonal communication during periods of theory of mind. Finally, relatively decreased activations in the amygdala during remote interpersonal interaction suggest less emotional engagement in these contexts compared with face-to-face environments. These differences are noteworthy and need to be explored further to understand how social cognition differs when the partners are not face-to-face.

## 8. Discussion

Remote interpersonal communication has changed the concept and definitions of social reward, as the implications differ greatly when the partners are separated by distance or through a screen. For the first time in history, individuals are spending more time communicating with others through virtual mechanisms than face-to-face. This phenomenon is yet to be fully understood, and the long-term effects remain unknown. We hypothesized, based on evidence, that the brain’s social reward networks are more frequently activated with increased use of remote interpersonal communication. This review discussed the neural mechanisms of social connection and how it is crucial for survival; we urge researchers to continue research on whether remote communication is enough to satisfy these needs. Although there are harmful effects from overuse of remote interpersonal communication, some individuals, especially those most heavily affected by the COVID-19 pandemic, rely on it to stay in touch with their loved ones. It would be interesting to research these special cases as well.

The opposite of social connection is social rejection, which also has important neural implications as studies have associated continued social rejection with reduced gray matter in the brain. Again, it is debated whether remote interpersonal communication provides more positive opportunities to connect with others and maintain relationships, or whether it primarily provides increased opportunities for social rejection. This controversy needs to be examined and further research is necessary. As stated, chronic social rejection can lead to voluntary or involuntary social isolation. This is known to damage the brain, especially when individuals are isolated from at a young age. A new phenomenon is arising through technology addictions in which individuals become physically socially isolated but are still communicating with others remotely. This needs to be researched further to determine whether or not remote interpersonal communication can satisfy human needs for social connection.

With increased technology use in recent years, as well as the COVID-19 pandemic’s mandatory lockdowns and its overall impact on society, social-cognitive neuroscientists have been presented with a new challenge. Human social isolation research has been very difficult to conduct in the past due to ethical concerns, yet the recent pandemic was able to provide a naturalistic setting for this research. Additionally, the younger generations are the first to be born into a world in which digital media is readily available. The impact of increased technology use or overuse starting from a young age has yet to be studied, especially its long-term effects. Pursuing these lines of research are likely to provide critical insights into the neural mechanisms of social cognition.

The purpose of this review is to highlight the ways in which social cognition is evolving both at the practical and neural levels due to remote interpersonal communication. We urge researchers to conduct research on the issues raised in this review. Most urgently, the field needs longitudinal research that leverages recent historical events and cultural/technological shifts to examine possible developmental changes in the social brain among those affected by the COVID-19 pandemic and technology overuse from a young age.

## Author contributions

MD and NL contributed to the conception and outline of the manuscript. MD wrote the first draft of the manuscript, created the figures, and led the literature review. NL contributed to literature review and wrote sections of the manuscript. Both authors contributed to the manuscript revision and approved the submitted version.
