# Supplementary material for: Social cognitive neuroscience in the digital age
Source: Front Hum Neurosci. 2023 May 30;17:1168788. doi: 10.3389/fnhum.2023.1168788 (PMC10265515; doi:10.3389/fnhum.2023.1168788)
Supplement: Supplementary file 1 [file Data_Sheet_1.PDF]

Supplementary Material

Social Cognitive Neuroscience in the Digital Age

Margaret M. Doheny*, Nichole R. Lighthall

*** Correspondence:** Margaret M. Doheny: Margaret.doheny@ucf.edu

Appendix A

Appendix A contains the citations for **Figure 1.**

Academic Dictionaries and Encyclopedias. (n.d.). Telegraph. In Academic Dictionaries and Encyclopedias. https://universalium.en-academic.com/209293/telegraph

Academic Dictionaries and Encyclopedias. (n.d.). Telephone. In Academic Dictionaries and Encyclopedias. https://universalium.en-academic.com/209322/telephone

Alba, A. (2016, March 31). Apple turns 40: Here's a timeline of how the tech giant evolved throughout the years. New York Daily News. https://www.nydailynews.com/news/national/apple-turns-40-timeline-tech-giant-evolution-article-1.2581048

Apple Watch. (2023, February 16). In Wikipedia; https://en.wikipedia.org/wiki/Apple_Watch#:~:text=The%20Apple%20Watch%20was%20released,Watch%20as%20of%20December%202020.

Associated Press. (2021). About Us. AP. https://www.ap.org/about/#:~:text=The%20Associated%20Press%20is%20an,vital%20to%20the%20news%20business.

Bellis, M. (2019, October 4). History of mail and the postal system. ThoughtCo. https://www.thoughtco.com/history-of-mail-1992142

CDC. (2022, August 16). CDC Museum COVID-19 Timeline. Centers for Disease Control and Prevention. https://www.cdc.gov/museum/timeline/covid19.html#:~:text=January%2010%2C%202020,%2DnCoV)%20on%20its%20website.

Chenda Ngak. (2011, September 26). The evolution of telephones. Cbsnews.com; CBS News. https://www.cbsnews.com/pictures/the-evolution-of-telephones/

Education Week Staff. (2021, March 5). A Year of COVID-19: What It Looked Like for Schools. Education Week. https://www.edweek.org/leadership/a-year-of-covid-19-what-it-looked-like-for-schools/2021/03

Facetime (2023, January 29). In Wikipedia; Wikimedia Foundation. https://en.wikipedia.org/wiki/FaceTime

4.2 History of Newspapers. (2016, March 22). Umn.edu; University of Minnesota Libraries Publishing edition, 2016. https://open.lib.umn.edu/mediaandculture/chapter/4-2-history-of-newspapers/

History of Phones. (2015). Odu.edu. https://www.cs.odu.edu/~tkennedy/cs300/development/Public/M01-HistoryOfPhones/index.html

McFadden, C. (2020, July 2). A Chronological History of Social Media. Interestingengineering.com; Interesting Engineering. https://interestingengineering.com/culture/a-chronological-history-of-social-media

Radiotelegraphy | communications | Britannica. (2023). In Encyclopædia Britannica. https://www.britannica.com/technology/radiotelegraphy

Tang, M. (2020, October 27). The History of Email: From 1970 till the present day - Exabytes Enterprise. Exabytes Enterprise. https://www.exabytes.cloud/blog/history-of-email/

The Daguerreotype Medium | Articles and Essays | Daguerreotypes | Digital Collections | Library of Congress. (2015). The Library of Congress. https://www.loc.gov/collections/daguerreotypes/articles-and-essays/the-daguerreotype-medium/#:~:text=Louis%2DJacques%2DMand%C3%A9%20Daguerre%20invented,Academy%20of%20Sciences%20in%20Paris.

Videophone | telephone | Britannica. (2023). In Encyclopædia Britannica. https://www.britannica.com/technology/videophone
